# Reassessment of *Allantonectria*, phylogenetic position of *Thyronectroidea*, and *Thyronectria caraganae* sp. nov

**DOI:** 10.1007/s11557-016-1218-4

**Published:** 2016-09

**Authors:** Hermann Voglmayr, Olexander Yu. Akulov, Walter M. Jaklitsch

**Affiliations:** 1 Division of Systematic and Evolutionary Botany, Department of Botany and Biodiversity Research, University of Vienna, Rennweg 14, 1030 Wien, Austria; 2 V. N. Karazin Kharkiv National University, Maidan Svobody 4, 61022 Kharkiv, Ukraine; 3 Institute of Forest Entomology, Forest Pathology and Forest Protection, Department of Forest and Soil Sciences, BOKU-University of Natural Resources and Life Sciences, Hasenauerstraße 38, 1190 Wien, Austria

**Keywords:** Ascomycota, Hypocreales, Nectriaceae, Phylogenetic analysis, Sordariomycetes, Taxonomy

## Abstract

The genus *Allantonectria* is synonymised with *Thyronectria*, based on morphological and molecular phylogenetic considerations. Investigations of types and fresh collections revealed that *Tubercularia concentrica* is an earlier name for *Sphaeria* (syn. *Allantonectria*) *miltina* and is thus combined in *Thyronectria*. *Allantonectria yuccae* is recognised as a distinct species and transferred to *Thyronectria*, as well as the recently described *A. zangii*. Descriptions and illustrations are provided for *T. concentrica* and *T. yuccae*. A recent collection of the North American *Thyronectroidea chrysogramma*, the generic type of *Thyronectroidea*, was studied with respect to morphology of its sexual morph in fresh condition and of its asexual morph produced in pure culture. Molecular phylogenies based on six loci (ITS and LSU regions of nuc rDNA, *act1*, *rpb1*, *rpb2*, *tef1* and *tub2* genes) place *T. chrysogramma* within *Thyronectria*, confirming synonymy of *Thyronectroidea* with *Thyronectria*, but remarkably a relationship to European species with green to brown spores (former genus *Mattirolia*) receives no support, and its closest relatives remain unclear. *Thyronectria caraganae* is described as a new species from herbarium specimens of *Caragana arborescens* collected in the Ukraine. It is characterised by morphology of the sexual morph and by DNA sequence data, which place it within the *T. austroamericana* - *T. rhodochlora* clade with high support. This is also supported by its morphology, specifically ascomata partly embedded within a stroma, muriform ascospores not budding within the ascus, becoming yellowish to rosy at maturity.

## Introduction

The genus *Thyronectria* was recently re-instated by Jaklitsch and Voglmayr (2014), as it takes precedence over the younger genus *Pleonectria*. Based on detailed morphological as well as molecular phylogenetic analyses, they synonymised *Mattirolia*, *Pleonectria* and *Thyronectroidea* with *Thyronectria* and combined all respective epithets in *Thyronectria*. This was also implemented by Lombard et al. (2015) in their overview of genera of the Nectriaceae.

Morphologically, the genus *Thyronectria* is mainly characterised by nectriaceous ascomata with a yellow scurf at the outer surface in combination with long, more or less persistent, apical paraphyses (Jaklitsch and Voglmayr 2014). Ascospores within the genus are highly diverse in size, shape, colour and septation; they can be ellipsoid, oblong, fusiform, globose, clavate or vermiform, with eusepta and/or distosepta, one- to several-septate or muriform, smooth or striate, and their colour can be hyaline, yellowish, rosy, green or brown. In several species, ascospores are budding in the ascus to produce oblong to allantoid, 1-celled, hyaline ascoconidia. Ecologically, several species have commonly been found in association with effete pyrenomycetes, indicating that they are fungicolous.

Subsequently, Checa et al. (2015) described two new *Thyronectria* species with olivaceous to green-brown muriform ascospores from Spain. In the phylogenetic analyses, the four known European *Thyronectria* species with green to brown muriform ascospores (*T. asturiensis*, *T. giennensis*, *T. pistaciae* and *T. roseovirens*) formed a highly supported subclade, together with *T. obscura*, which has hyaline muriform, budding ascospores.

The lack of fresh collections and cultures precluded the inclusion of the North American *Thyronectroidea chrysogramma*, which also has greenish to brown muriform ascospores, in molecular phylogenetic analyses. Based on morphological investigations, it was included in *Mattirolia* by Checa et al. (2013), while Jaklitsch and Voglmayr (2014) provided convincing arguments for a placement within *Thyronectria*. A recent Canadian collection from the type host, *Ulmus americana*, enabled us to document its sexual morph in fresh condition, to study its asexual morph produced in pure culture and to assess its phylogenetic position by DNA sequence data.

In the course of a revision of herbarium specimens at the V. N. Karazin Kharkiv National University, Ukraine (CWU), several specimens of an unidentified nectriaceous fungus were revealed on *Caragana arborescens*. All specimens were originally deposited at the M.G. Kholodny Institute of Botany, NAS of Ukraine, Kyiv (KW). Although their characters matched the genus *Thyronectria*, they could not be identified with the keys of Jaklitsch and Voglmayr (2014) and Checa et al. (2015). DNA sequence data subsequently obtained from ascomata confirmed an affiliation with *Thyronectria*, and following molecular phylogenetic and morphological analyses, it is here described as a new species.

The genus *Allantonectria* (Earle 1901) has been revealed as closely related to *Thyronectria* in molecular phylogenies with high support (Hirooka et al. 2012; Jaklitsch and Voglmayr 2014; Checa et al. 2015; Lombard et al. 2015). Hirooka et al. (2012) accepted *Allantonectria* as a separate monotypic genus, characterized by minute, one-celled allantoid to rod-shaped ascospores and growth on monocotyledonous hosts as diagnostic characters. In previous works (Höhnel and Weese 1910; Lowen 1991; Hirooka et al. 2012), the generic type of *Allantonectria*, *A. yuccae* from *Yucca* spp., was regarded as a synonym of *A. miltina*, which was described from *Agave americana*. However, none of these authors mentioned *Tubercularia concentrica*, although this species was considered to be the asexual morph of *A. miltina* in the older literature (e.g., Saccardo 1878, 1883) and which, in case of conspecificity, would take precedence due to priority.

The description of *A. zangii* from *Populus* sp. (Zeng and Zhuang 2012, 2013), a non-monocotyledonous host, and the lack of significant support for a sister group relationship of *Thyronectria* to *Allantonectria* in recent molecular phylogenies (Jaklitsch and Voglmayr 2014; Checa et al. 2015) called for a re-evaluation of the generic status of *Allantonectria*. We performed detailed morphological studies of *Allantonectria* specimens from *Agave* and *Yucca* spp. for comparison with *Thyronectria* and to evaluate the species status of *A. miltina* and *A. yuccae*. In addition, we examined the types and fresh collections of *Tubercularia concentrica* and *A. miltina* to investigate whether they are conspecific. Based on these investigations, we conclude that *T. concentrica* and *A. miltina* are synonymous, *A. yuccae* represents a distinct species and *Allantonectria* should be merged with *Thyronectria*.

## Materials and methods

### Morphological observations

Microscopic preparations were mounted in water, 3 % potassium hydroxide (KOH) or lactic acid (LA). Methods of microscopy included stereomicroscopy using a Nikon SMZ 1500 and Nomarski differential interference contrast (DIC) using the Zeiss Axio Imager.A1 compound microscope. Images and data were gathered using the Nikon DS-U2 or Zeiss Axiocam 506 color digital cameras and measured by using the NIS-Elements D v.3.0 or Zeiss ZEN Blue Edition softwares. For certain images of ascomata the stacking software Zerene Stacker v.1.04 (Zerene Systems, Richland, WA, USA) was used. Measurements are reported as maxima and minima in parentheses and the range representing the mean plus and minus the standard deviation of a number of measurements given in parentheses.

### Culture observations

Due to lack of fresh material of *Thyronectria caraganae*, no cultures could be obtained. Cultures of *T. chrysogramma* and *T. concentrica* were prepared and maintained as described previously (Jaklitsch 2009) except that 2 % malt extract agar (MEA) was used for isolation. Germinating ascospores/conidia were placed on MEA and CMD (CMA: Sigma, St Louis, MI, USA; supplemented with 2 % (w/v) D(+)-glucose-monohydrate) or 2 % malt extract agar (MEA; 2 % w/v malt extract, 2 % w/v agar-agar; Merck, Darmstadt, Germany). Cultures used for the study of asexual morph micro-morphology were grown on 2 % MEA or CMD at room temperature (RT; 22 ± 3 °C) under alternating 12 h daylight and 12 h darkness. Microscopic observations were made in tap water except where noted. The plates were sealed with laboratory film and incubated at room temperature. A culture of *T. chrysogramma* was deposited at CBS-KNAW Fungal Biodiversity Centre, Utrecht, The Netherlands (CBS).

### DNA extraction, PCR and sequencing

The extraction of genomic DNA, PCR and sequencing of segments of six loci, i.e., the nuc rDNA region encompassing the internal transcribed spacers 1 and 2, along with the 5.8S, and the D1-D2 domains of the 28S (ITS-LSU), α-actin (*act1*) gene, RNA polymerase II subunit 1 (*rpb1*) and subunit 2 (*rpb2*) genes, translation elongation factor 1-α (*tef1*) gene, and β-tubulin (*tub2*) gene, was performed as reported in Jaklitsch and Voglmayr (2014), but using the newly designed primers dRPB2-5f (5′ GAYACNGAYGAYCGWGAYCAYTTYGG 3′) and dRPB2-7r (5′ AANCCCATDGCYTGYTTDCCCAT 3′) for amplification and sequencing of *rpb2*. As no living cultures were available for *T. caraganae*, DNA was directly extracted from ascomata using the protocol described in Voglmayr and Jaklitsch (2011).

### Phylogenetic analyses

**Fig. 1:**
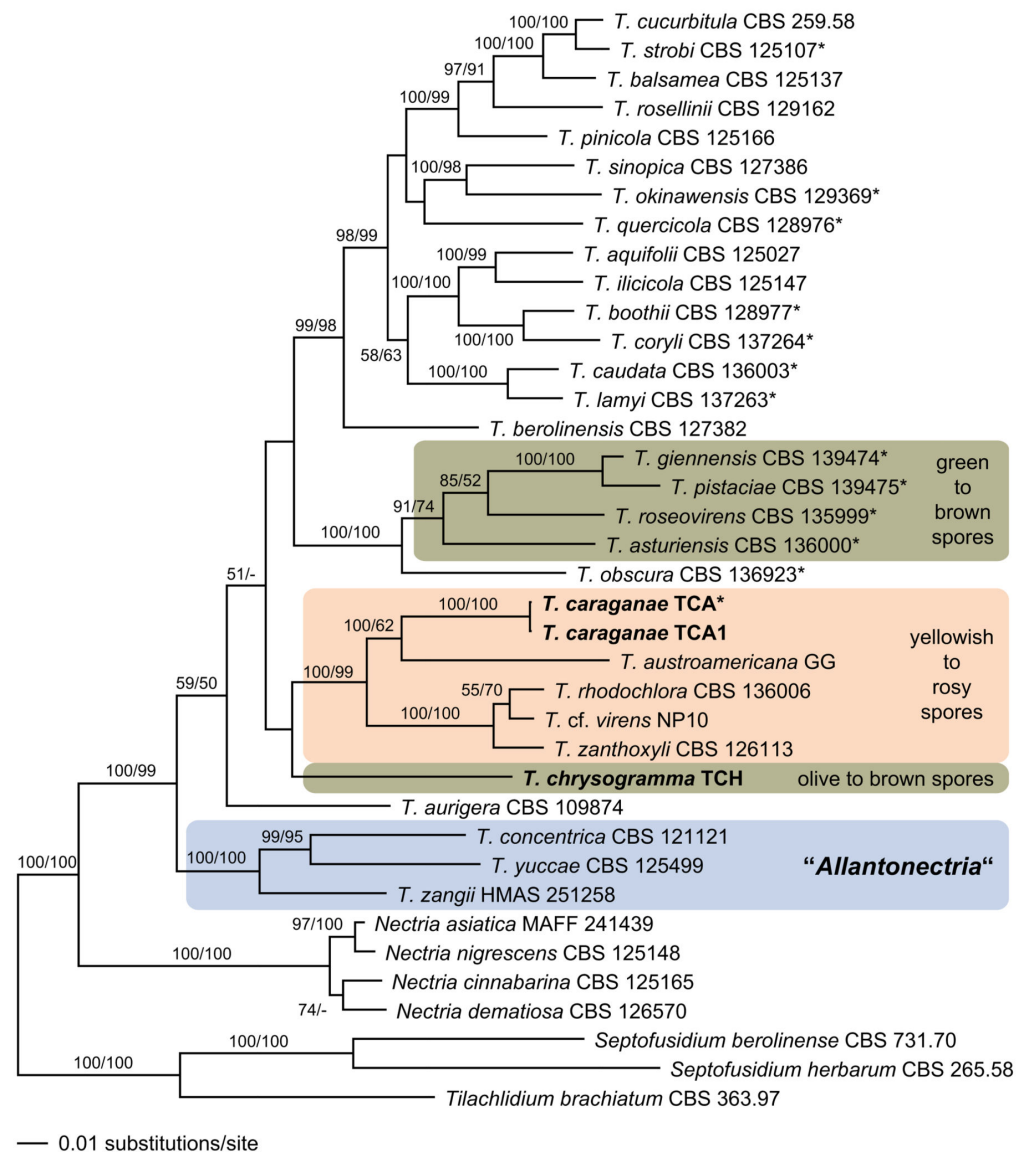
Phylogram of the best maximum likelihood tree (lnL = −49176.5326) revealed by RAxML from an analysis of the combined matrix of *Thyronectria*, showing the phylogenetic position of *T. chrysogramma* and *T. caraganae* (in *bold*). ML and MP bootstrap support above 50% are given above or below the branches. Strain or herbarium numbers are given following the taxon names; holo-, neo- or epitype strains/specimens are marked by an *asterisk* (*). The tree was rooted with *Septofusidium berolinense*, *S. herbarum* and *Tilachlidium brachiatum* (Tilachlidiaceae) according to Lombard et al. (2015)

A single accession of each *Thyronectria* species was included in the phylogenetic analyses. The accessions were selected according to availability of markers and, if possible, ex-type sequences were used (marked with an asterisk in Fig. 1). In addition, four *Nectria* species were included, and *Septofusidium berolinense*, *S. herbarum* and *Tilachlidium brachiatum* (Tilachlidiaceae) were selected as outgroup according to Lombard et al. (2015). Available sequences were downloaded from GenBank; details on the sequences used in the phylogenetic analyses are provided in Table 1.

All alignments were produced with the server version of MAFFT (www.ebi.ac.uk/Tools/mafft or http://mafft.cbrc.jp/alignment/server/), checked and refined using BioEdit v.7.0.4.1 (Hall 1999). To reveal the phylogenetic position of the newly sequenced *Thyronectria* species, the newly generated sequences were aligned with the GenBank sequences. The resulting combined sequence matrix contained 6292 alignment positions from six gene regions (630 from *act1*, 541 from ITS and 807 from LSU, 706 from *rpb1*, 1192 from *rpb2*, 1308 from *tef1* and 1108 from *tub2*).

Maximum likelihood (ML) analyses were performed with RAxML (Stamatakis 2006) as implemented in raxmlGUI 1.3 (Silvestro and Michalak 2012) using the ML+ rapid bootstrap setting and the GTRGAMMAI substitution model with 1000 bootstrap replicates. Substitution model parameters were calculated separately for the different gene regions included in the combined analyses.

Maximum parsimony (MP) analyses were performed with PAUP v.4.0a147 (Swofford 2002), using 1000 replicates of heuristic search with random addition of sequences and subsequent TBR branch swapping (MULTREES option in effect, steepest descent option not in effect). All molecular characters were unordered and given equal weight; analyses were performed with gaps treated as missing data; the COLLAPSE command was set to MAXBRLEN. Bootstrap analysis with 1000 replicates was performed in the same way, but using 5 rounds of random sequence addition and subsequent TBR branch swapping during each bootstrap replicate, with the COLLAPSE command set to MINBRLEN.

## Results

### Cultures and sequences of sexual and asexual morphs of *Thyronectria concentrica*

Growth characteristics of cultures of *T. concentrica* obtained from the presumed asexual morph matched those obtained from ascospores, and the asexual morph produced in pure cultures from ascospores and conidia was identical. The ITS-LSU sequences of cultures isolated from the sexual and asexual morphs were identical to GenBank sequences of *Allantonectria miltina* from *Agave americana*.

### Molecular phylogeny

Of the 6292 characters of the combined matrix, 1857 were parsimony informative (142 in *act1*, 119 in ITS, 108 in LSU, 304 in *rpb1*, 485 in *rpb2*, 393 in *tef1* and 306 in *tub2*). The phylogram of the best ML tree (lnL = −49176.5326) obtained by RAxML is shown in Fig. 1. The MP analysis revealed a single tree of length 9703 (not shown), which is similar to the ML tree except for a different position of *T. chrysogramma* (see below), a sister group relationship of *Nectria dematiosa* to the other three *Nectria* species, and a placement of *T. quercicola* following next to *T. berolinensis*. Except for minor differences, tree topologies agree well with those of Jaklitsch and Voglmayr (2014) and Checa et al. (2015).

The *Thyronectria*–*Allantonectria* clade is highly supported in both ML and MP analyses (Fig. 1), *Allantonectria* receives maximum support, but *Thyronectria* receives only low support. The ML and MP analyses reveal different phylogenetic positions of *T. chrysogramma*. In the ML analyses, *Thyronectria chrysogramma* is placed basal to the highly supported clade containing species with yellowish to rosy spores, but this placement does not receive ML bootstrap support. In the MP analysis, *T. chrysogramma* is the most basal taxon of the *Allantonectria*–*Thyronectria* clade, however, again without bootstrap support. *Thyronectria caraganae* is placed in the highly supported clade containing species with yellowish to rosy spores; sister group relationship to *T. austroamericana* receives low (MP) or high (ML) support.

### Taxonomy

**Fig. 2:**
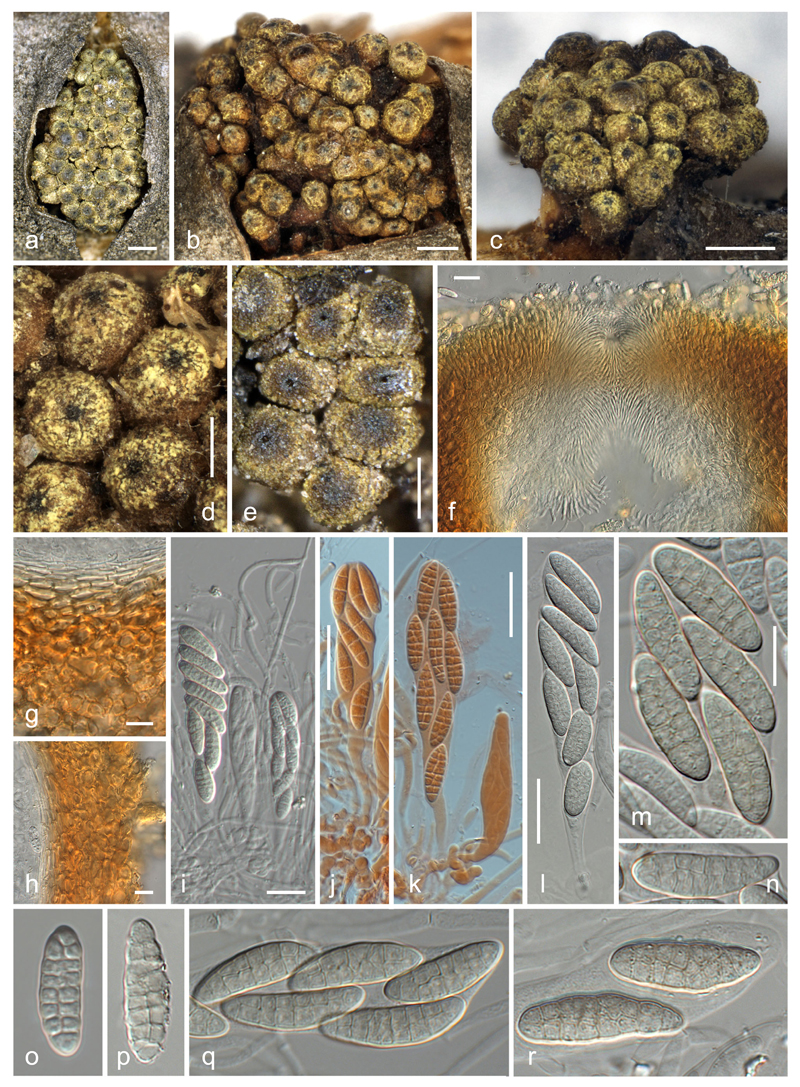
*Thyronectria caraganae*. **a**–**e** Stromata/ascomata. **f**–**h** Peridium in vertical section (**f** ostiolar region; **g** base; **h** side; in 3 % KOH). **i**–**l** Asci with ascospores (**i** with apical paraphyses; **j**, **k** immature; **l** mature; **i, l** in 3 % KOH; **j, k** in Congo Red). **m**–**r** Ascospores (**o** immature; all in 3 % KOH). **a,** **e**–**k, o, p** WU 35938; **b** WU 35941; **c, d** WU 35940; **m, n, q, r** WU 35939. *Scale bars* (**a**–**c**) 500 μm; (**d, e**) 200 μm; (**f**–**l**) 20 μm; (**m**–**r**) 10 μm

***Thyronectria caraganae*** Voglmayr, Akulov & Jaklitsch, sp. nov. Fig. 2.

MycoBank: MB 817627.

Etymology: referring to its host, *Caragana arborescens*.

Stromata erumpent from bark, surrounded by bark flaps or projecting above the bark level; stromatic tissue surrounding ascomata that are densely aggregated in numbers of up to ca. 120 in 1.2–3.2(−4.4) mm long, 0.9–2.8(−3.1) mm wide (*n* = 19) and 0.7–1.5 mm high, rounded or elongated clusters. Ascomata globose to obovoid, not becoming cupulate upon drying, (275–)320–420(−530) μm diam in surface view (*n* = 92), ca. 330–430 μm high when dry, varying in colour from yellow to dull olive brown, turning orange-red in 3 % KOH, entirely covered by greenish-yellow scurf of minute amorphous particles when young; scurf vanishing around the ostiolar region with age. Peridium 30–45 μm thick at the base, ca. 45–70 μm at the sides, consisting of a thin (ca. 10– 15 μm) hyaline to yellowish inner layer of strongly compressed, elongate (4.5–)6–10(−12) μm (*n* = 20) long cells and an outer ca. 20–55 μm thick layer of thick-walled, compressed cells (6.5–)7.5–14(−19) μm (*n* = 40) diam, tending to be more isodiametric outward, pigmented from the outside to the inside dark orange/yellow-orange/yellow in 3 % KOH, releasing yellow pigment in KOH; without a distinct pH-dependent colour change in KOH and LA. Ostiolar region (30–)43–71(−90) μm diam in surface view (*n* = 68) when dry, slightly papillate or flat-umbilicate, darker than the main part of the ascoma, dark olive-brown to black. Ostiole periphysate, ca. 16–27 μm wide in surface view when dry. Periphyses pointed, short, 15–30(−40) μm long, 2–3 μm wide, projecting into the ostiole and slightly downward. Apical paraphyses numerous, indistinct in KOH, embedded in a slime matrix when immature, hanging down to the base of the asci, richly branched and anastomosing, 2–4 μm wide. Asci clavate, (78–)85–103(−118) × (12–)15–19(−22) μm (*n* = 57), with variable stipe and undifferentiated apex, containing 8 obliquely uniseriate or biseriate ascospores. Ascospores ellipsoid or oblong, straight or curved, (14.3–)18–24 (−28.4) × (4.8 –)6.3–7.7(−9.2) μm, l/w = (1.9–)2.5–3.5(−4.4) (*n* = 272), muriform, with (5–)7(−10) transverse and (0–)1(−2) longitudinal, less commonly oblique septa, hyaline and often more oblong when immature, turning yellowish to rosy at full maturity, smooth, not budding in the ascus.

Asexual morph: Not observed.

Distribution: Eastern Europe, only known from the Ukraine.

Ecology: On dead corticated branches of *Caragana arborescens*; possibly fungicolous and associated with *Cucurbitaria caraganae*.

Holotype: Ukraine, Mykolaiv district, Berezansky area, Tashine, on *Caragana arborescens*, 16 May 1990, L.V. Smyk (WU 35938 holotype, KW 7033/8583 and CWU (MYC) AS374 isotypes; ex-holotype sequences KX514384 (ITS-LSU rDNA), KX514381 (*act1*), KX514389 (*rpb1*), KX514395 (*tef1*), KX514398 (*tub2*)).

Other material studied: Ukraine, Donets’k district, Volnovachasky area, Ol’shanka, on *Caragana arborescens*, 31 July 1986, L.V. Smyk (KW 7419/7853, CWU (MYC) AS430, WU 35939); Dnipropetrovs’k district, Pjatihatsky area, along motorway Dnipropetrovs’k - Pjatihatky, on *Caragana arborescens*, 10 Oct. 1973, L.V. Smyk (KW 7418/7852, CWU (MYC) AS429, WU 35941); Zaporizhzhya district, Akimovsky area, Bogatyrskoe forest, on *Caragana arborescens*, 19 Jan. 1972, M.F. Smitskaya (KW 7417/7851, CWU (MYC) AS428, WU 35940).

Notes: The lack of fresh material precluded pure culture isolation, but sequencing of DNA extracted from ascomata revealed sequences of five of the six loci used in the phylogenetic analyses. *Thyronectria caraganae* is well characterised by the dull olive-brown ascomata surrounded by stromatic tissue covered by a greenish-yellow scurf, which are usually densely aggregated in large numbers; the hyaline to yellowish or rosy elongate-ellipsoid muriform ascospores with usually seven indistinct transverse septa and typically a single longitudinal septum; and its host, *Caragana arborescens*. No asexual morph was observed on the herbarium specimens available for study. *Thyronectria caraganae* is closely related to *T. austroamericana*, which also occurs on fabaceous hosts and also forms large clusters of ascomata partly immersed in a stroma covered by a bright yellow scurf, but differs by ascospores of different size, shape and septation, by ascomata of dull olive brown colour and by the lack of a conspicuous zythiostroma-like asexual morph in nature (Hirooka et al. 2012; Jaklitsch and Voglmayr 2014).

**Fig. 3:**
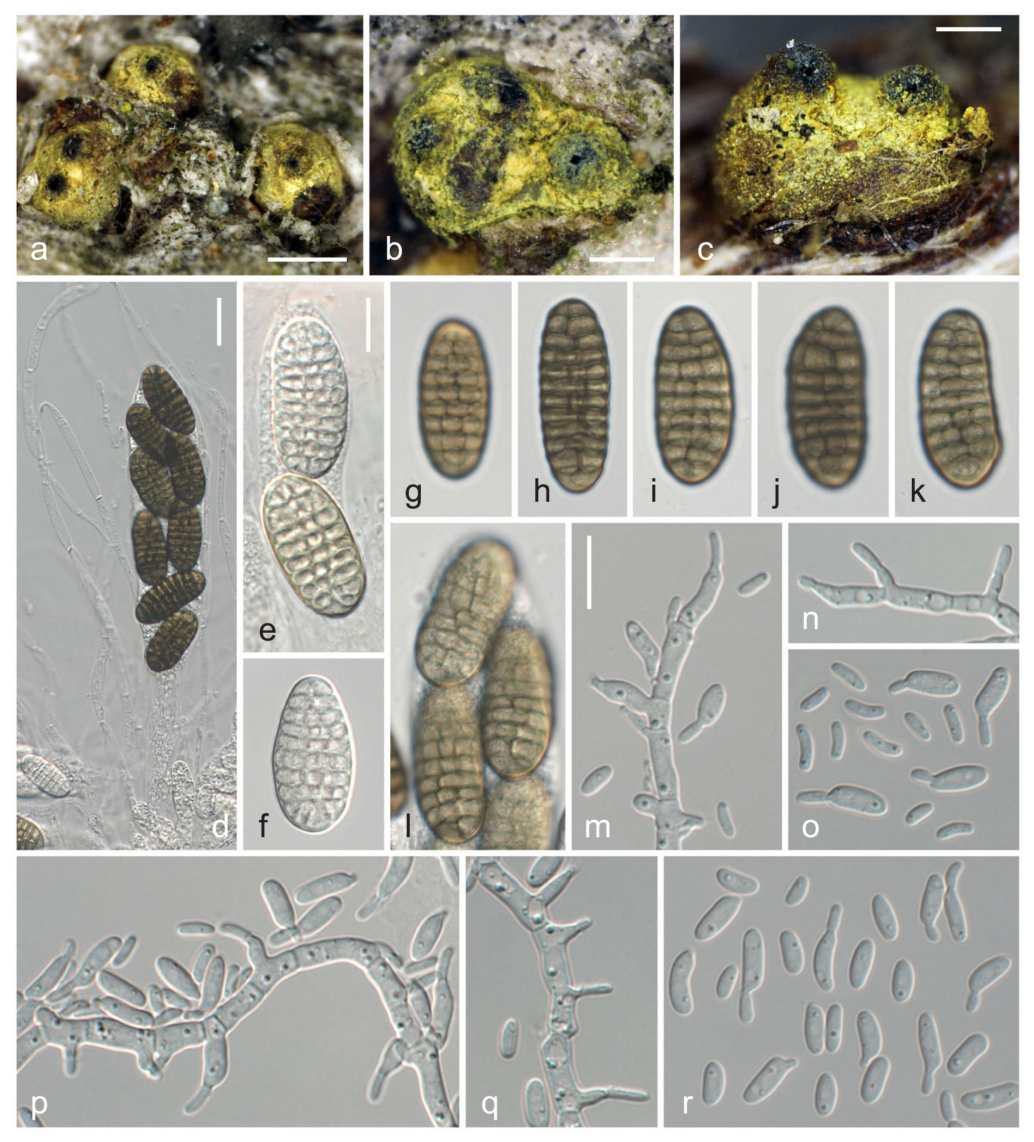
*Thyronectria chrysogramma* (WU 35942 = TCH). **a**–**c** Stromata. **d** Apical paraphyses and ascus. **e**–**l** Vital ascospores (**e, f** immature; **g**–**l** mature). **m, n, p, q** Effuse conidiation on hyphae showing pegs and phialides (MEA, RT, 2 days). **o, r** Conidia (MEA, RT, 2 days); **d**–**r** in water. *Scale bars* (**a**) 500 μm; (**b, c**) 200 μm; (**d**) 20 μm; (**e**–**r**) 10 μm

***Thyronectria chrysogramma*** Ellis & Everh., Proc. Acad. nat. Sci. Philad. 42: 245. 1890. Fig. 3.

Synonyms. *Mattirolia chrysogramma* (Ellis & Everh.) Sacc., Syll. fung. (Abellini) 9: 993. 1891.

*Nectria chrysogramma* (Ellis & Everh.) Rossman, Mem. N. Y. bot. Gdn 49: 259. 1989.

*Thyronectroidea chrysogramma* (Ellis & Everh.) Seaver, Mycologia 1: 206. 1909.

Ascomata immersed and erumpent from bark, subglobose, 0.3–0.5 mm diam, scattered or aggregated, individually surrounded by yellowish stromatic tissue, sometimes immersed in soft, pulvinate, erumpent stromata to ca. 1 mm diam, covered with yellow to yellow-green scurf. Peridium reddish, weakly reacting in 3 % KOH and LA. Ostiolar area black, ostioles periphysate. Apical paraphyses numerous, richly branched, 1.5–5 μm wide. Asci (152–)150–195(−213) × (25–)27–33(−37) μm (*n* = 10), spore bearing part (102–)106–135(−145) μm (*n* = 14) long, clavate, containing 8 biseriately arranged ascospores; apex undifferentiated. Ascospores (24–)28.7–34.5(−37) × (10–)12.5–15(−17.3) μm, l/w = (1.6–)2.1–2.5(−3.2) (*n* = 100), first hyaline and oblong, turning yellowish and finally olive-brown, becoming medium to reddish-brown in old herbarium specimens, and oblong or ellipsoid, with 4–9(−11) transverse and (1–)2–3 conspicuously closely inserted longitudinal eusepta, smooth.

Cultures and asexual morph: On MEA colony up to 58 mm diam after 20 days at room temperature; surface cream, with cottony tufts of aerial hyphae, turning rosy from the centre due to conidial masses; reverse cream to pale orange. On CMD colony up to 62 mm diam after 20 days at room temperature; white, conidiation sparse. Conidiation on phialides or hyphal pegs. Phialides (4.5–)6.5–11(−16) × (2.3–)2.5–3.2(−3.5) μm (*n* = 33), formed directly on hyphae, mostly solitary, terminal or lateral, flask-shaped to cylindrical. Pegs on hyphal cells mostly close to the septa, (0.9–)1.5–4.3(−6.8) × (1.1–)1.3–2.7(−5.2) μm (*n* = 51). Conidia mainly formed on pegs, (3.8–)5.0–7.0(−9.6) × (1.3–)1.8–2.7(−3.4) μ m, l/w = (1.9–)2.3–3.1(−4.3) (*n* = 221), ellipsoid to allantoid, unicellular, hyaline, smooth, eguttulate or with few minute, often subterminal guttules, budding.

Distribution: North America

Ecology: Only known from bark of *Ulmus americana*, possibly fungicolous.

Specimen examined: Canada, Ontario, Ottawa, on bark of *Ulmus americana*, 24 Dec 2015, J. Mack (WU 35942, culture TCH = CBS 141087).

Notes: We provide here an extended description of Jaklitsch and Voglmayr (2014), which was based on the holo- and paratype, to include characters of the sexual morph in fresh condition, as well as culture characteristics and morphology of the asexual morph. The characters of the fresh collection agree well with the type material, which was illustrated in Checa et al. (2013; as *Mattirolia chrysogramma*) and Jaklitsch and Voglmayr (2014). As in type material, most perithecia are not aggregated into larger stromatic groups but occur solitarily and scattered in the fresh collection. Phylogenetic data place this species within the genus *Thyronectria*, confirming the conclusions based on morphological analyses. However, a closer relationship to the European *Thyronectria* species with green to brown ascospores does not receive support, and its closest relatives within *Thyronectria* remain unclear.

**Fig. 4:**
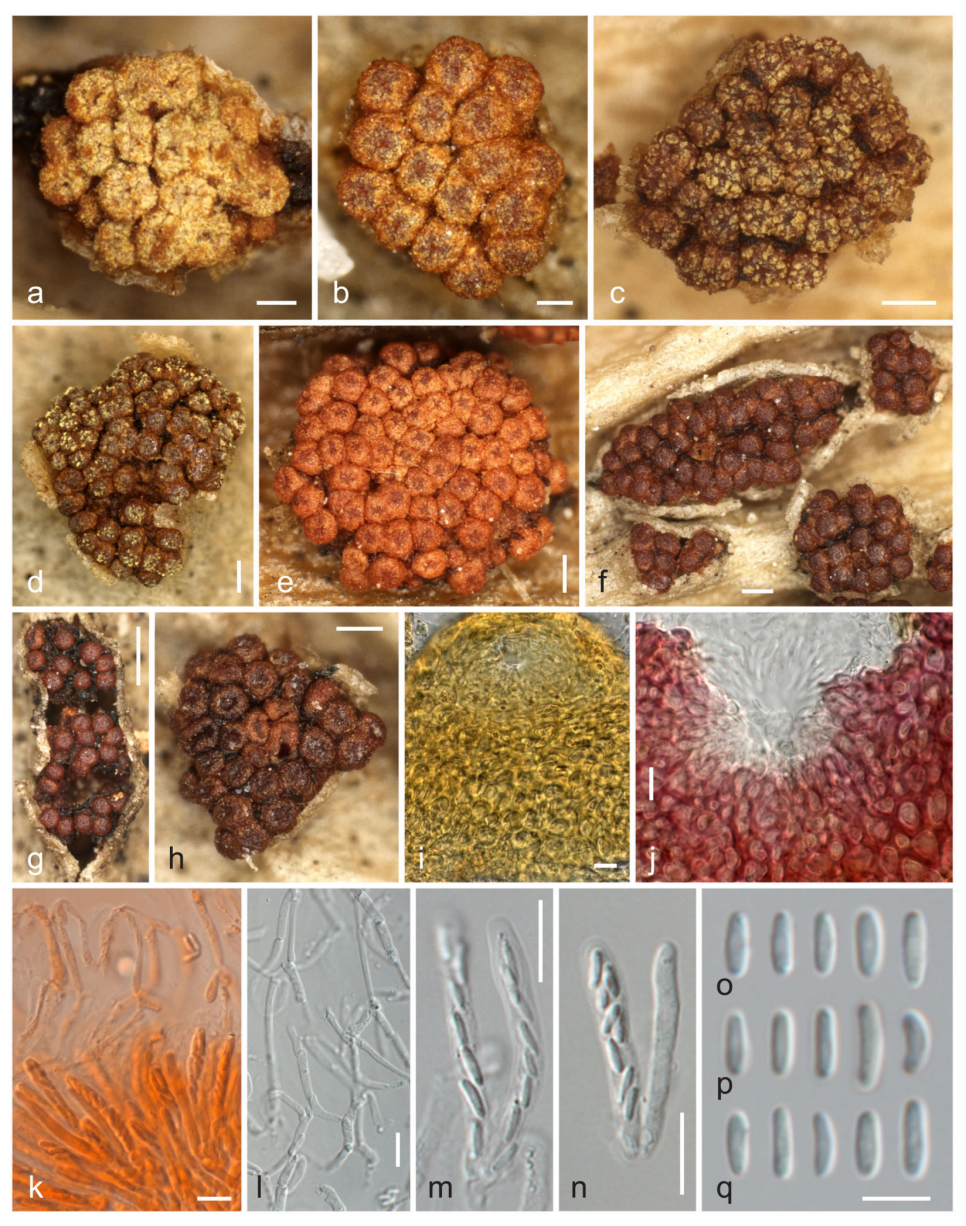
*Thyronectria concentrica*, sexual morph. **a**–**h** Stromata/ascomata (**a**–**e** with yellow scurf, **h** cupulate). **i, j** Peridium around ostiolar region in LA (**i**) and 3 % KOH (**j**). **k**. Asci with ascospores and apical paraphyses (in Congo Red). **l**. Network of apical paraphyses. **m, n** Asci. **o**–**q** Ascospores (all in 3 % KOH except where noted). **a, d** W 1973-00252; **b, j, l**–**n, p** W 1912-3086; **c, e, f, o** W 1914-10028; **g, i** W 1914-9263; **h, q** W 1904-4875); **k** W 1914-9263. *Scale bars* (**a, b**) 100 μm; (**b**–**f, h**) 200 μm; (**g**) 500 μm; (**i**–**n**) 10 μm; (**o**–**q**) 5 μm

**Fig. 5:**
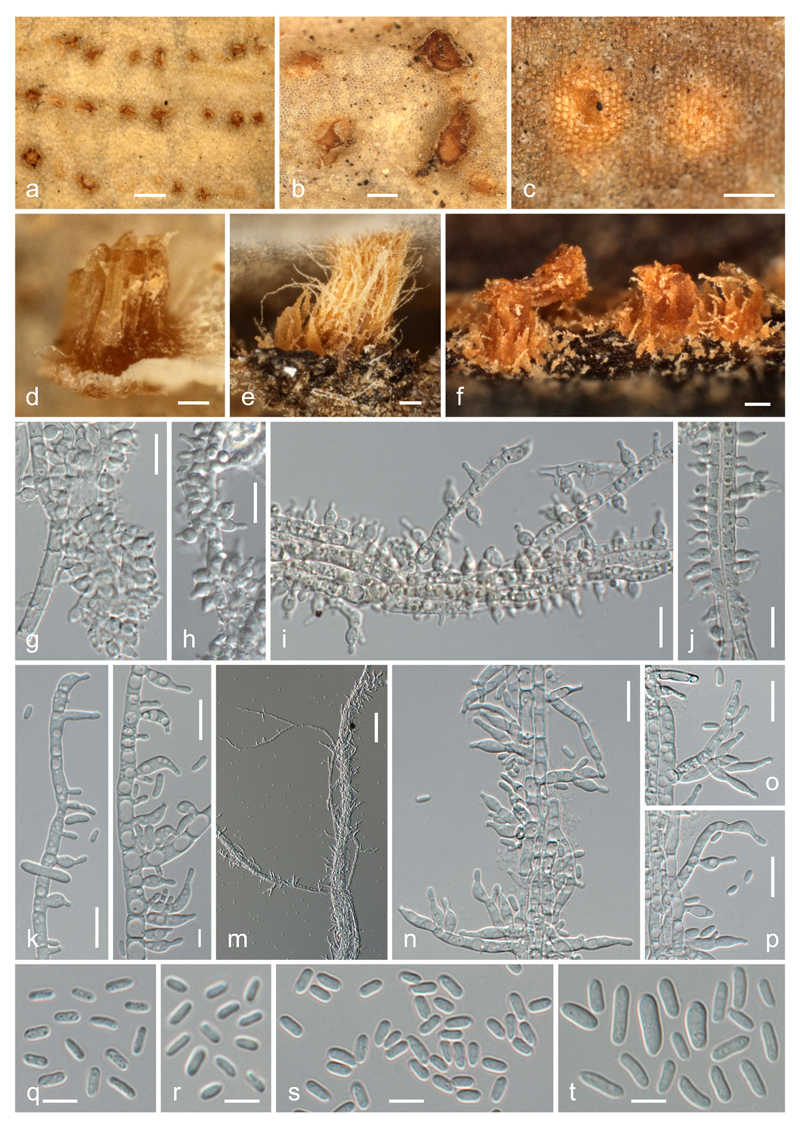
*Thyronectria concentrica*, asexual morph. **a**–**f** Synnematous conidiomata on natural substrates (**a**–**c** in surface view, **d**–**f** in side view after rupture of the host epidermis). **g**–**j** Squash mounts of synnematous conidiophores with lateral phialides in 3 % KOH (**g, h**) and water (**i, j**). **k,** **l** Effuse conidiation on hyphae showing pegs and phialides (MEA, RT, 3 days). **n**–**p** Conidiophores and phialides on aerial hyphal strands (MEA, RT, 7 days). **q**–**t** Conidia [**q, r** from natural substrates, **s** from aerial strands (MEA, RT, 7 days), **t** from effuse sporulation (MEA, RT, 3 days)] (all in water except where noted). **a, b, g, r** K(M) 201845; **c, e, f, i, j, q** WU 35943; **d** K(M) 203419; **h** K(M) 203420; **k**–**p, s, t** WU 35944. *Scale* *bars* (**a**) 10 mm; (**b**) 300 μm; (**c**–**f**) 100 μm; (**g**–**l,** **n**–**p**) 10 μm; (m) 50 μm; (**q**–**t**) 5 μm

***Thyronectria concentrica*** (Mont. & Fr.) Voglmayr & Jaklitsch, comb. nov. Figs. 4, 5.

MycoBank: MB 817628

Basionym. *Tubercularia concentrica* Mont. & Fr., in Montagne, Ann. Sci. Nat., Bot., sér. 2 6: 28 (1836).

Synonyms. *Sphaeria miltina* Durieu & Mont., in Durieu, Expl. Sci. Algérie, Bot. I, Flore d’Algérie: 477. 1848 [1846–1849].

*Allantonectria miltina* (Mont.) Weese, in Höhnel & Weese, Ann. mycol. 8(3): 467 (1910).

*Nectria miltina* (Mont.) Mont., Syll. gen. sp. crypt. (Paris): 225 (1856).

*Nectriella miltina* (Mont.) Sacc., Michelia 1(no. 3): 278 (1878).

Stromata erumpent through leaf epidermis, up to 2.5 mm diam, pulvinate, tubercular, orange to dark reddish brown when dry, inside yellow, KOH+ red, LA+ yellow, pseudoparenchymatous, cells forming *textura angularis* to *t. globulosa*, intergrading with ascomatal wall. Ascomata superficial on the hypostroma, commonly surrounded by epidermis flaps, scattered to aggregated in groups of (2–)5–75, subglobose to globose, sometimes with a depressed apical region upon drying, 145–245 μm high, (145–)162–198(−240) μm diam (*n* = 133), varying in colour from orange, red to dark reddish-brown, apical region darker when young, concolorous with age, KOH+ deep to blackish red, LA+ yellow, entirely covered by yellow scurf of minute amorphous particles when young. Peridium 25–50 μm thick, consisting of a thin (6–13 μm) inner layer of elongate, thin-walled, hyaline cells forming a *textura prismatica* and an outer ca. 20–40 μm thick layer of thick-walled cells 5–14 μm diam, forming a *textura globulosa* or *t. angularis*, of pigmented walls about 1.5 μm thick with a distinct pH-dependent colour change, violaceous-red in 3 % KOH, yellow in LA. Ostiolar region flat-umbilicate, usually darker than the main part of the perithecium when young. Ostiole periphysate, ca. 10–17 μm wide. Periphyses numerous, ca. 2 μm thick. Apical paraphyses numerous, indistinct in KOH, hanging down to the base of the asci, richly branched and anastomosing, consisting of inflated cells readily disarticulating upon pressure, 2–4.5 μm wide. Asci narrowly clavate, (26–)28–35(−41) × (3.0–)3.5–4.5(−5.0) μm (*n* = 35), with variable stipe and undifferentiated apex, 8-spored; ascospores biseriate above, uniseriate below. Ascospores allantoid to short-cylindrical, rounded at both ends, (3.3–)4.3–5.5(−7.3) × (0.9–)1.2–1.6(−2.0) μm, l/w = (2.5–)3.1–4.2(−5.9) (*n* = 595), aseptate, hyaline, smooth, not budding in the ascus.

### Asexual morph on natural substrates

Synnematous, developing below the epidermis, orange to orange brown, in young stage translucent through epidermis as circular to elongate pale yellow to bright orange spots 0.3–1.3 mm diam, becoming free after rupturing of the epidermis, forming a fissured column of tightly packed septate yellowish, 2.5–5.5 μm thick hyphae. Phialides (4.3–)4.5–6.8(−11.2) × (2.3–)2.9–3.8(−4.9) μm (*n* = 62), densely aggregated on outermost hyphae of the column, mostly solitary, lateral, flask-shaped, sometimes curved. Conidia (2.7–)3.5–4.2(−4.9) × (1.1–)1.4–1.7(−2) μm, l/w = (1.8–)2.2–2.9(−3.7) (*n* = 201), ellipsoid to slightly allantoid, unicellular, hyaline, smooth, eguttulate or with few minute, often subterminal guttules.

### Asexual morph in pure culture

On MEA colony up to 46 mm diam after 7 days at room temperature; surface cream, with tufts of aerial mycelium appressed to hyphal strands, turning pink from the centre due to conidial masses; reverse cream, conidiation starting on superficial substrate hyphae, later mainly on aerial hyphal strands. On CMD colony up to 59 mm diam after 7 days at room temperature; white, aerial hyphae sparse, conidiation mainly on superficial substrate hyphae. Conidiophores emerging as fasciculate side branches on strands of parallel hyaline aerial hyphae, short, 2–4.3 μm wide, simple, with few verticils of 2–3 branches. Conidiation on phialides or hyphal pegs. Phialides (4.5–)6.0–9.2(−10.5) × (2.5–)3.0–3.7(−4.3) μm (*n* = 40), formed directly on hyphae or on short conidiophores, mostly solitary or in whorls of 2–3, terminal or lateral, flask-shaped to cylindrical, straight, curved, mostly inequilateral. Pegs on hyphal cells (0.8–)1.1–3.2(−5.2) × 1–1.7(−2.2) μm (*n* = 22). Conidia on surface hyphae (3.0–)4.5–7(−9.7) × (1.3–)1.5–2.5(−) μm, l/w = (1.8–)2.4–3.4(−4.8) (*n* = 103), on aerial hyphal strands (3.2–)3.5–4.3(−5.5) × (1.3–)1.6–1.8(−2) μm, l/w = (1.8–)2.1–2.5(−3.2) (*n* = 100), ellipsoid to slightly allantoid, unicellular, hyaline, smooth, eguttulate.

Distribution: Africa, Mediterranean Europe, North America.

Ecology: On dead leaves of *Agave* spp.

Typification: France, Perpignan, on *Agave americana*, ex Herb. C. Montagne (K(M) 201845!, lectotype of *Tubercularia concentrica* here designated; K(M) 203419!, K(M) 203420! isotypes). Algeria, near Mustapha, on *Agave americana*, 22 Dec. 1839, M. C. Durieu (PC 0723442!, lectotype of *Sphaeria miltina* here designated). Algeria, without place, date and collector, Herb. C. Montagne (PC 0723441!, PC 0723446!, isotypes of *Sphaeria miltina*).

Other material studied (all on dead leaves of *Agave americana*): Algeria, Alger, 22 Dec. 1839, M. C. Durieu (PC 0723443); ibid., 21 Dec. 1839, M. C. Durieu (PC 0723439); ibid., 15 Jan. 1840, M. C. Durieu (PC 0723438). Greece, Corfu, Mon repos, Apr. 1912, K. Rechinger (W 1914-10028); ibid., same date and collector, in Rehm, Ascomyc. 1962b (W 1914-9263). Italy, Südtirol, Arco-Meran, 1911, W. Dittrich-Kalkhoff, in Rehm, Ascomyc. 1962 (W1912-3086, W1973-00252); at shore of Lago di Garda, without date, Duby, in Rabenhorst, Herb. Mycol. II 631 (W1923-2100); Napoli, Botanical Garden, Cesati, in Rabenhorst, Fungi Eur. Exs. 1828a (sexual morph, labelled *Nectria miltina*), 1828b (asexual morph, labelled *Tubercularia concentrica*) (W 2016-02661); Sicilia, Messina, Motta Camastra, Gole Alcantara Parco Botanico e Geologico, 19 June 2016, H. Voglmayr & W. Jaklitsch (WU 35949). Montenegro, Ulcinj, 10 Apr. 1903, F. Bubak, in Vestergren, Micromyc. Rar. Sel. Praec. Scand. 829 (W 1904-4875). Spain, Islas Canarias, La Gomera, Juego de Bolas, 20 Mar. 2016, H. Voglmayr & I. Greilhuber (WU 35943; culture from asexual morph ALLA); La Gomera, Chipude, 23 Mar. 2016, H. Voglmayr & I. Greilhuber (WU 35944; culture from sexual morph ALLM).

Notes: This species has been commonly known as *Allantonectria miltina* (see Hirooka et al. 2012). In older literature (e.g., Saccardo 1878, 1883), *Tubercularia concentrica* has been given as the asexual morph of *A. miltina*, but Lowen (1991) and Hirooka et al. (2012) did not mention the name in their synonymies. Hirooka et al. (2012) did not record an asexual morph from natural substrates. In one of our recent collections, we found a synnematous asexual morph developing below the host epidermis, and its connection with the sexual morph was subsequently proven by pure cultures and sequence data. Investigation of three isotype specimens of *Tubercularia concentrica* revealed that they fully match the asexual morph of our recent collection, and we here select the well-developed specimen K(M) 201845 as lectotype. As *Tubercularia concentrica* is older than *Sphaeria miltina*, the epithet *concentrica* has to be used, which is here combined in *Thyronectria* (see discussion below).

The asexual morph produced on SNA has been described as trichoderma-like by Hirooka et al. (2012). In our cultures on 2 % MEA, conidiation started on surface hyphae similar to other *Thyronectria* species, but was later primarily present on the hyphal strands of the aerial mycelium.

*Thyronectria concentrica* appears to be widespread in the Mediterranean where its main host, *Agave americana*, is commonly cultivated and naturalised. Like its host, it is assumed to originate from North America. Based on sequence data (Fig. 1), we do not accept synonymy of *T. concentrica* and *T. yuccae*, which mainly differ in their hosts (*Agave* vs. *Yucca*; see below) and by different ascospore sizes. In addition, *T. concentrica* commonly has more ascomata per stroma (up to 75 vs. up to 30 in *T. yuccae*). We include here a short description and illustration of *T. concentrica* in addition to the detailed description by Hirooka et al. (2012, under *Allantonectria miltina*) to facilitate comparison with *T. yuccae* and to describe the apical paraphyses and the asexual state on the natural substrate, which have not yet been documented for this species. As in all *Thyronectria* species we have investigated (Jaklitsch and Voglmayr 2014; Checa et al. 2015), apical paraphyses are numerous and more or less persistent, but they are delicate, easily disarticulating and therefore only seen after careful ascoma preparation preferably in KOH (herbarium specimens) or water (fresh samples).

The collection data (year and collector) of the specimen given as holotype of *Sphaeria miltina* by Hirooka et al. (2012) do not agree with the protologue and cannot represent a type. Lowen (1991) designated a specimen from Herb. Montagne deposited in PC as lectotype, but the specimen labels of the two extant collections from Herb. Montagne do not bear the collection data she gave in the text, and the packages contain no information about lectotypification. In addition, the specimens were rearranged on a new sheet, with one fragment in PC 0723446 and four fragments in PC 0723441, whereas Lowen (1991) mentions the presence of two fragments in the specimen she examined, so it is unclear which specimen she selected. We here select specimen PC 0723442 from Herb. Durieu as lectotype of *Sphaeria miltina*, which is the only authentic specimen from PC on which Mustapha, the place mentioned in the protologue, is given on the original label. The two specimens from Herb. Montagne without place and date are considered to represent isotypes.

**Fig. 6:**
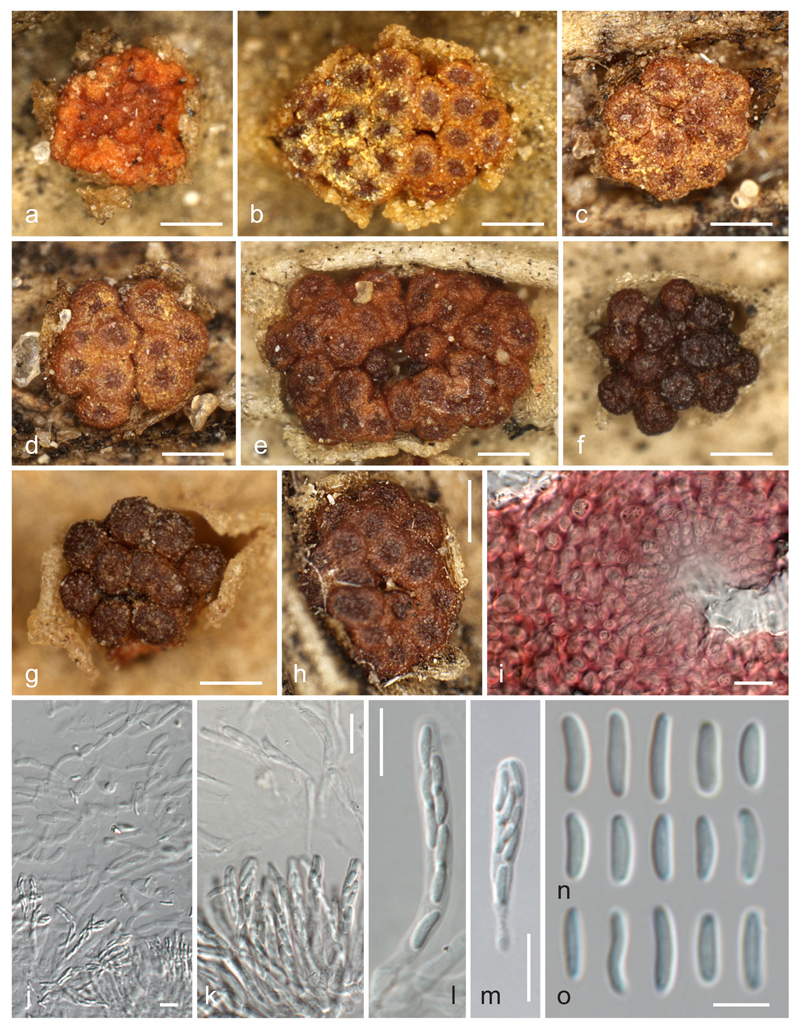
*Thyronectria yuccae*. **a** Young hypostroma. **b**–**h** Stromata/ascomata (**b**–**d** with yellow scurf): **i**. Peridium around ostiolar region in 3 % KOH. **j, k** Asci with ascospores and apical paraphyses. **l, m** Asci. **n, o** Ascospores (all in 3 % KOH except where noted). **a, b, e, j, m, o** W 1981-08871; **c, d, h, i, k, n** W1912-3086; **f** W 1971-14238; **g** W 1901-6035 (isotype). *Scale bars* (**a**–**h**) 200 μm; (**i**–**m**) 10 μm; (**n, o**) 5 μm

***Thyronectria yuccae*** (Earle) Voglmayr & Jaklitsch, comb. nov. Fig. 6.

MycoBank: MB 817629

Basionym. *Allantonectria yuccae* Earle in Greene, Plant. Bak. 2(1): 11 (1901).

Stromata erumpent through leaf epidermis, up to 1.2 mm diam, pulvinate, tubercular, orange to dark reddish-brown when dry, inside yellow, KOH+ red, LA+ yellow, pseudoparenchymatous, cells forming *textura angularis* to *t. globulosa*, intergrading with the ascomatal wall, 4–12 μm diam. Ascomata superficial on the hypostroma, commonly surrounded by epidermis flaps, scattered to aggregated in groups of (2)5–20(30), subglobose to globose, sometimes with a depressed apical region upon drying, 140–170 μm high, (125–)155–190(−230) μm diam (*n* = 121), varying in colour from orange, red to dark reddish-brown, apical region commonly slightly darker when young, concolorous with age, KOH+ deep to blackish-red, LA+ yellow, entirely covered by yellow scurf of minute amorphous particles when young. Peridium 50–60 μm thick, consisting of a thin (ca. 10–15 μm) inner layer of elongate, thin-walled, hyaline cells forming a *textura prismatica* and an outer ca. 40–45 μm thick layer of thick-walled cells 3–10 μm diam, forming a *textura globulosa* or *t. angularis*, of pigmented walls about 1–1.5 μm thick with a distinct pH-dependent colour change, violaceous-red in 3 % KOH, yellow in LA. Ostiolar region flat-umbilicate, commonly darker than the main part of the perithecium when young. Ostiole periphysate, ca. 10–20 μm wide. Periphyses numerous, ca. 2 μm thick. Apical paraphyses numerous, indistinct in KOH, extending to the base of the asci, richly branched and anastomosing, consisting of inflated cells easily disarticulating upon pressure, 1–4.5 μm wide. Asci narrowly clavate, (27–)30–41(−48) × (3.7–)4–5(−5.8) μm (*n* = 34), with variable stipe and undifferentiated apex, 8-spored; ascospores biseriate above, uniseriate below. Ascospores allantoid to short-cylindrical, rounded at both ends, (4.5–)5.5–7.3(−9.6) × ( 1. 1 –)1.4–1.8 (−2. 3) μm, l/w = (2.7–)3.5–4.7(−6.2) (*n* = 301), aseptate, hyaline, smooth, not budding in the ascus.

Asexual morph on natural substrates: Not observed.

Distribution: North and Central America.

Ecology: On dead leaves of *Yucca* spp.

Type: USA, Colorado, Hermosa, on *Yucca* sp., March 1899, C.F. Baker (W 1901-6035!, isotype)

Other material studied: USA, California, Riverside County, near San Jacinto, Soboba Hot Springs, on dead leaves of *Yucca schidigera*, 6 Mar. 1955, H. E. Parks 9007, in California Fungi 1127 (W 1963-7695, W 1973-06127); Colorado, Boulder, on dead leaves of *Yucca* sp., Aug. 1950, F. Petrak 10127 (W 1971-14238); Leyden, on dead leaves of *Yucca glauca*, 2 May 1910, E. Bethel, (W 1981-08871); Nebraska, Valentine, on dead leaves of *Yucca* sp., 23 Feb. 1898, C.S. Shear, (W 1974-22484).

Notes: This species is the generic type of *Allantonectria* (Earle 1901), described from *Yucca* sp. collected in Colorado (USA). In combining the older *Sphaeria miltina*, which was described from *Agave americana* collected in Algeria, in *Allantonectria*, Höhnel and Weese (1910) synonymised *A. yuccae* with the older *A. miltina*, evidently based on similar ascospore sizes. This synonymy was also accepted by Hirooka et al. (2012), who cited specimens from both host genera, but only included sequences from a European accession from *Agave americana* in their phylogenies. Recently, sequences from an accession from *Yucca elata* collected in Arizona (USA) became available (Lombard et al. 2015), which differ substantially from the European collections of *T. concentrica* (see also Fig. 1). We therefore re-investigated specimens from both host genera, including an isotype of *A. yuccae* deposited in W, and found that *A. yuccae* has larger ascospores compared to *T. concentrica* ((4.5–)5.5–7.3(−9.6) × (1.1–)1.4–1.8(−2.3) vs. (3.3–)4.3–5.5(−7.3) × (0.9–)1.2–1.6(−2.0) μm). We therefore consider *A. yuccae* to represent a species distinct from *T. concentrica*, and it is here combined in *Thyronectria*.

***Thyronectria zangii*** (Z.Q. Zeng & W.Y. Zhuang) Voglmayr & Jaklitsch, comb. nov.

MycoBank: MB 817630

Basionym. *Nectria zangii* Z.Q. Zeng & W.Y. Zhuang, Mycotaxon 120: 69 (2012).

Synonym. *Allantonectria zangii* (Z.Q. Zeng & W.Y. Zhuang) Z.Q. Zeng & W.Y. Zhuang, Mycosystema 32(2): 294 (2013).

Holotype. China, Beijing, Donglingshan, alt. 1150 m, on branches of *Populus* sp., 20 July 2011, Z.Q. Zeng & H.D. Zheng 7684 (HMAS 251247, not seen).

Notes: *Thyronectria zangii* closely resembles *T. concentrica* and *T. yuccae* in its minute unicellular rod-shaped ascospores, but differs by thinner perithecial walls, narrower asci, and occurrence on twigs of *Populus* (Zeng and Zhuang 2012). Ascospore size of *T. zangii* (3.5–5.5(−6) × 0.9–1.2(−1.4) μm) is similar to that of *T. concentrica* revealed in the present study. Like for *T. yuccae*, no asexual morph has been recorded for *T. zangii* from natural substrates.

## Discussion

Molecular phylogenetic analyses confirm the conclusions of Jaklitsch and Voglmayr (2014) that *Thyronectroidea chrysogramma* belongs to *Thyronectria*. However, it is not contained within the clade comprising European species with green to brown ascospores (formerly classified in *Mattirolia*), but its phylogenetic position within *Thyronectria* remains uncertain. In the MP analyses, it is placed basal to the *Allantonectria*–*Thyronectria* lineage, a position which does not receive support, whereas in the ML analyses it is placed in a basal position of the clade containing the species with yellowish to rosy spores, again without support.

While the *Thyronectria*–*Allantonectria* clade and *Allantonectria* are highly supported, the genus *Thyronectria* receives only low support in both MP and ML analyses (Fig. 1). It should be noted that, with the addition of species and markers, the medium to high internal support of a sister group relationship of *Allantonectria* to *Thyronectria* as revealed by Hirooka et al. (2012), has decreased significantly in subsequent publications (Jaklitsch and Voglmayr 2014; Checa et al. 2015), which casts doubt on the status of *Allantonectria* as a separate genus. With the addition of *A. zangii* from *Populus* (Zeng and Zhuang 2013), monocotyledonous substrates are no longer distinctive for *Allantonectria*, and the only remaining difference between the two genera lies in the small, unicellular allantoid to rod-shaped ascospores of *Allantonectria*. Considering the highly diverse ascospore sizes, shapes, colours and septations prevailing in *Thyronectria*, we do not regard ascospore characters alone to be suitable for generic delimitation, and it appears justified to include *Allantonectria* in *Thyronectria*. The presence of persistent apical paraphyses and the asexual morph in pure culture, which were revealed in the present study, also correspond to *Thyronectria*. In the light of this morphological and molecular phylogenetic evidence, we argue for inclusion of *Allantonectria* in *Thyronectria*, and we formally combine the three accepted species of *Allantonectria* in *Thyronectria*.

Based on culturing and sequencing of recent collections as well as type studies, we were able to confirm that *Tubercularia concentrica* is the asexual morph of *A. miltina*, and we here provide an extended description of the asexual morph on natural substrates for the first time. Lowen (1991) and Rossman et al. (1999) mentioned *Tubercularia* sp. as asexual morph; however, without giving any description. Hirooka et al. (2012) did not find an asexual morph on natural substrates, probably due to its development within decaying leaves prior to ascomata, whereas they expected it to be pycnidial. At first sight, the synnematous conidiomata of *T. concentrica* do not fit the concept of *Thyronectria*, which was considered to be characterised by pycnidial conidiomata (Hirooka et al. 2012, under *Pleonectria*). However, not all *Thyronectria* species have pycnidial conidiomata, but some like *T. roseovirens* show effuse sporulation on natural substrates (Jaklitsch and Voglmayr 2014). Also, the “trichoderma”-like conidiation on aerial hyphal strands in pure culture are not unique for *T. concentrica*, but similar conidiation is also observed, e.g., in *T. asturiensis* (Jaklitsch and Voglmayr 2014). Therefore, although pycnidial asexual morphs are common within *Thyronectria*, they cannot be considered diagnostic for the whole lineage.

*Thyronectria caraganae* is revealed as the closest relative of *T. austroamericana* within the clade comprising species with yellowish to rosy ascospores. Apart from spore colour, species within this clade are characterised by ascomata surrounded by stromatic tissue (Hirooka et al. 2012; Jaklitsch and Voglmayr 2014). In addition, all species have muriform ascospores, which do not bud within the ascus or only rarely do so. All these characters are also present in *T. caraganae*.

Remarkably, *T. caraganae* is so far only known from the Ukraine, although its host, *Caragana arborescens*, is widely planted throughout Europe at road banks and between fields as a shrub preventing soil erosion by wind. However, it appears to be much rarer than its host, and may be confined to areas of continental climate. Despite pronounced search, the fungus could not be found in eastern Austria where its host is commonly cultivated. In eastern Austria, *Caragana arborescens* is frequently colonised by another nectriaceous fungus with similar ecology, *Stromatonectria caraganae* (Jaklitsch and Voglmayr 2011a), which may be the reason for the absence of *Thyronectria caraganae* in this area. However, the latter is an example for the lack of biodiversity inventories, even in well-studied Central Europe. Despite its conspicuous bright red stromata, *S. caraganae* had not been recorded for more than 100 years after its description, although it not only persisted in its type locality, a botanical garden (Jaklitsch and Voglmayr 2011a), but also turned out to be very common on *Colutea arborescens* and *Caragana arborescens*, and sometimes *Laburnum anagyroides* (all members of the Fabaceae), in eastern Austria after more detailed investigations. The detection of *T. caraganae* on a widely distributed host once again demonstrates the need of biodiversity studies on corticolous ascomycetes in Europe, of which a substantial amount of species is still poorly known and undescribed (e.g., Voglmayr and Jaklitsch 2008, 2011, 2014; Jaklitsch and Voglmayr 2011a, b, 2014; Voglmayr et al. 2012, 2016; Jaklitsch et al. 2013, 2014, 2015, 2016; Checa et al. 2015).

## Figures and Tables

**Table 1:** Isolates and accession numbers used in the phylogenetic analyses; those in bold were isolated/sequenced in the present study

Species	Isolate No.	Herbarium No.^a^	Substrate/Host	Country	GenBank accession numbers						
					*act1*	ITS	LSU	*rpb1*	*rpb2*	*tef1*	*tub2*
*N. asiatica*	MAFF 241439	BPI 879972	unid. dead bark	Japan	HM484505	HM484701	HM484563	*–*	JQ014140	*–*	HM484604
*N. cinnabarina*	CBS 125165	BPI 879981	*Aesculus* sp.	France	HM484503	HM484548	HM484562	HM484577	JQ014125	HM484527	HM484606
*N. dematiosa*	CBS 126570	BPI 749337	unid. dead bark	USA	HM484502a	HM484557	HM484561	HM484576	JQ014144	HM484534	HM484603
*N. nigrescens*	CBS 125148	BPI 871083	unid. dead twigs	USA	HM484618	HM484707	HM484720	HM484781	JQ014123	HM484672	HM484806
*Septofusidium berolinense*	CBS 731.70				KM231250	KM231841	KM231722	KM232274	KM232417	KM231978	KM232112
*S. herbarum*	CBS 265.58		*Urtica dioica*	UK	KM231251	KM231842	KM231723	KM232275	KM232418	KM231979	KM232113
*Thyronectria aquifolii*	CBS 125027	WU 30360	*Ilex aquifolium*	UK	KJ570663	HM534891	HM534891	KJ570715	HM534881	HM534870	KJ570638
*T. asturiensis*	CBS 136000	WU 32124	*Quercus ilex*	Spain	KJ570664	KJ570690	KJ570690	KJ570716	KJ570741	KJ570760	KJ570639
*T. aurigera*	CBS 109874	BPI 841465	*Fraxinus excelsior*	France	HM484511	HM484551	HM484573	HM484586	–	HM484521	HM484600
*T. austroamericana*	GG	WU 32664	*Gymnocladus dioicus*	Austria	KJ570665	KJ570691	KJ570691	KJ570717	KJ570742	KJ570761	KJ570640
*T. balsamea*	CBS 125137	NCSU	*Abies fraseri*	USA	JF832454	JF832599	JF832729	JF832805	JQ014142	JF832561	JF832849
*T. berolinensis*	CBS 127382	WU 30361	*Ribes sanguineum*	Austria	KJ570666	HM534893	HM534893	KJ570718	HM534883	HM534872	KJ570641
*T. boothii*	CBS 128977	BPI 881052	*Picea abies*	Slovakia	JF832475	JF832617	JF832755	JF832796	–	JF832552	JF832871
*T. caraganae*	**TCA**	WU 35938	*Caragana arborescens*	Ukraine	**KX514381**	**KX514384**	**KX514384**	**KX514389**	–	**KX514395**	**KX514398**
*T. caraganae*	**TCA1**	WU 35939	*Caragana arborescens*	Ukraine	**KX514382**	**KX514385**	**KX514385**	**KX514390**	–	**KX514396**	**KX514399**
*T. caudata*	CBS 136003	WU 32130	*Berberis cretica*	Greece	KJ570667	KJ570692	KJ570692	KJ570719	KJ570743	KJ570762	KJ570642
*T. chrysogramma*	**TCH, CBS 141087**	WU 35942	*Ulmus americana*	Canada	**KX514383**	**KX514386**	**KX514386**	**KX514391**	**KX514392**	**KX514397**	**KX514400**
*T. concentrica*	CBS 121121	BPI 878442	*Agave americana*	Italy	HM484514	HM484547	HM484572	HM484587	KM232409	HM484524	HM484609
	**ALLA**	WU 35943	*Agave americana*	Spain	–	**KX514387**	**KX514387**	–	–	–	–
	**ALLM**	WU 35944	*Agave americana*	Spain	–	**KX514388**	**KX514388**	–	**KX514393**	–	–
*T. coryli*	CBS 137264	WU 32129	*Corylus avellana*	Austria	KJ570669	KJ570693	KJ570693	KJ570721	KJ570744	KJ570763	KJ570644
*T. cucurbitula*	CBS 259.58		*Pinus sylvestris*	Netherlands	GQ505974	HM484541	GQ505998	GQ506028	JQ014131	HM484530	HM484592
*T. giennensis rotundifolia*	CBS 139474	AH 47011	*Quercus ilex* ssp. *rotundifolia*	Spain	KR057941	KR057943	KR057943	KR057945	KR057947	KR057949	KR057951
*T. ilicicola*	CBS 125147	BPI 880698	*Ilex aquifolium*	UK	HM484506	HM484538	HM484565	HM484579	–	HM484522	HM484590
*T. lamyi*	CBS 137263	WU 32159	*Berberis vulgaris*	Austria	KJ570672	KJ570695	KJ570695	KJ570723	KJ570746	KJ570765	KJ570647
*T. miltina*	CBS 121121	BPI 878442	*Agave americana*	Italy	HM484514	HM484547	HM484572	HM484587	KM232409	HM484524	HM484609
*T. obscura*	CBS 136923	WU 32142	*Tamarix tetrandra*	Austria	KJ570673	KJ570699	KJ570699	KJ570724	KJ570747	KJ570766	–
*T. okinawensis*	CBS 129369, MAFF 241410	BPI 881058, TUA-TPP-h92	*Castanopsis* sp.	Japan	JF832451	JF832674	JF832751	JF832827	–	JF832585	JF832878
*T. pinicola*	CBS 125166	BPI 881059	*Pinus sylvestris*	Germany	HM484508	HM484540	HM484567	HM484580	–	HM484528	HM484591
*T. pistaciae*	CBS 139475	AH 45402	*Pistacia lentiscus*	Spain	KR057942	KR057944	KR057944	KR057946	KR057948	KR057950	KR057952
*T. quercicola rotundifolia*	CBS 128976	BPI 871328	*Quercus ilex* ssp. *rotundifolia*	Spain	JF832450	JF832624	JF832743	JF832831	KM232411	JF832581	JF832880
*T. rhodochlora*	CBS 136006	WU 31656	*Acer campestre*	Austria	KJ570678	KJ570704	KJ570704	KJ570729	**KX514394**	KJ570771	KJ570651
*T. rosellinii*	CBS 129162	BPI 881066	*Abies balsamea*	USA	JF832474	JF832614	JF832739	JF832820	–	JF832578	JF832870
*T. roseovirens*	CBS 135999	WU 32154	*Laburnum alpinum*	Italy	KJ570683	KJ570709	KJ570709	KJ570734	KJ570754	KJ570776	KJ570656
*T. sinopica*	CBS 127386	WU 30364	*Hedera helix*	Austria	KJ570689	HM534900	HM534900	KJ570740	HM534890	HM534879	KJ570662
	CBS 125107	NY	*Pinus strobus*	USA	JF832467	JF832605	JF832725	JF832813	–	JF832569	JF832861
T. strobi											
*T.* cf. *virens*	NP10	WU 33426	*Ostrya carpinifolia*	France	KM225678	KM225684	KM225684	KM225689	–	KM225696	KM225701
*T. yuccae*	CBS 125499		*Yucca elata*	USA	KM231247	KM231836	KM231717	KM232270	HQ897730	KM231974	KM232107
*T. zangii*	HMAS 251258	HMAS 251258	*Populus* sp.	China	JX843456	JN997424	JX843458	JX843452	–	JX843454	JN997421
*T. zanthoxyli*	CBS 129157	BPI 881069	unid. dead bark	USA	JF832510	JF832627	JF832753	JF832833	–	JF832590	JF832884
*Tilachlidium brachiatum*	CBS 363.97		*Agaricus* sp.	France	KM231248	KM231838	KM231719	KM232271	KM232414	KM231975	KM232109

a *AH* Universidad de Alcalá, Madrid, Spain, *BPI* U.S. National Fungus Collections USDA-ARS MD USA, *CBS* Centraalbureau voor Schimmelcultures, Utrecht, The Netherlands, *HMAS* Institute of Microbiology, Chinese Academy of Sciences, Beijing, China, *MAFF* MAFF Genebank, National Institute of Agrobiological Sciences, Ibaraki, Japan, *NCSU* The Mycological Herbarium, North Carolina State University, NC, USA, *NY* The New York Botanical Garden, New York, USA, *TUA-TPP-h* Yuuri Hirooka, Tropical Plant Protection Lab Herbarium, Tokyo University of Agriculture, Tokyo, Japan, *WU* Herbarium of the University of Vienna, Austria
